# Distinct expression of synaptic NR2A and NR2B in the central nervous system and impaired morphine tolerance and physical dependence in mice deficient in postsynaptic density-93 protein

**DOI:** 10.1186/1744-8069-4-45

**Published:** 2008-10-14

**Authors:** Wen-Jinn Liaw, Xu-Guang Zhu, Myron Yaster, Roger A Johns, Estelle B Gauda, Yuan-Xiang Tao

**Affiliations:** 1 Department of Anesthesiology and Critical Care Medicine, Johns Hopkins University School of Medicine, Baltimore, Maryland 21205, USA; 2 Department of Anesthesiology, Tri-Service General Hospital/National Defense Medical Center, Taipei 114, Taiwan, Republic of China; 3 Department of Pediatrics, Division of Neonatology, Johns Hopkins University School of Medicine, Baltimore, Maryland 21287, USA

## Abstract

Postsynaptic density (PSD)-93, a neuronal scaffolding protein, binds to and clusters *N*-methyl-D-aspartate receptor (NMDAR) subunits NR2A and NR2B at cellular membranes *in vitro*. However, the roles of PSD-93 in synaptic NR2A and NR2B targeting in the central nervous system and NMDAR-dependent physiologic and pathologic processes are still unclear. We report here that PSD-93 deficiency significantly decreased the amount of NR2A and NR2B in the synaptosomal membrane fractions derived from spinal cord dorsal horn and forebrain cortex but did not change their levels in the total soluble fraction from either region. However, PSD-93 deficiency did not markedly change the amounts of NR2A and NR2B in either synaptosomal or total soluble fractions from cerebellum. In mice deficient in PSD-93, morphine dose-dependent curve failed to shift significantly rightward as it did in wild type (WT) mice after acute and chronic morphine challenge. Unlike WT mice, PSD-93 knockout mice also showed marked losses of NMDAR-dependent morphine analgesic tolerance and associated abnormal sensitivity in response to mechanical, noxious thermal, and formalin-induced inflammatory stimuli after repeated morphine injection. In addition, PSD-93 knockout mice displayed dramatic loss of jumping activity, a typical NMDAR-mediated morphine withdrawal abstinence behavior. These findings indicate that impaired NMDAR-dependent neuronal plasticity following repeated morphine injection in PSD-93 knockout mice is attributed to PSD-93 deletion-induced alterations of synaptic NR2A and NR2B expression in dorsal horn and forebrain cortex neurons. The selective effect of PSD-93 deletion on synaptic NMDAR expression in these two major pain-related regions might provide the better strategies for the prevention and treatment of opioid tolerance and physical dependence.

## Introduction

Opioid drugs such as morphine are a class of powerful analgesics used for treating many forms of acute and chronic pain. However, their chronic use has been limited by undesirable side effects such as tolerance, abnormal pain sensitivity, and physical dependence [1,2]. These undesirable symptoms are believed to be related to neuronal plasticity in the central nervous system (CNS). Several lines of research have shed light on molecular and cellular mechanisms that underlie the development of opioid analgesic tolerance and dependence [3-5]. Pharmacological blockade of NMDA receptors (NMDARs) or targeted disruption of NMDAR subunit NR2 genes significantly attenuates symptoms of opioid tolerance and physical dependence, implicating involvement of NMDARs in the development of opioid-induced neuronal plasticity [6-8]. However, the molecular mechanisms underlying NMDAR-dependent synaptic plasticity during the development of opioid tolerance and physical dependence are unclear.

PSD (post synaptic density)-93, also named chapsyn (channel-associated protein of synapses)-110, is one of a growing superfamily of PDZ-domain-containing proteins shown to physically link proteins together into macromolecular structures [9,10]. PSD-93 was identified to have structural similarity with three other PDZ-domain-containing proteins, PSD-95/SAP (synapse-associated protein) 90 [11,12], SAP102 [12,13], and SAP97/hdlg [14,15]. These proteins are generically referred to as membrane-associated guanylate kinases (MAGUKs) and contain three tandem PDZ domains (PDZ1-3) at the N-terminal side, an Src homology region 3 domain in the middle, and a guanylate kinase-like domain at the C-terminal end. PDZ domains of MAGUKs are motifs of ~90 amino acid repeats that have recently been recognized to mediate protein-protein interactions. Studies using the yeast two-hybrid system revealed that the PDZ domains of PSD-93 specifically bind to the C-termini of NMDAR subunits NR2A and NR2B [10]. The deletion of PDZ domains from PSD-93 not only disrupts interaction between NR2A/NR2B and PSD-93, but also reduces NMDAR clustering at cellular membranes *in vitro *[10]. Targeted disruption of the PSD-93 gene reduces NMDAR-mediated postsynaptic function in dorsal horn and forebrain cortex and attenuated NMDAR-mediated persistent pain [16]. However, a recent study reported that PSD-93 knockout (KO) mice displayed normal NMDAR-mediated postsynaptic response in hippocampal neurons [17]. It appears that the roles of PSD-93 in synaptic NMDAR targeting and NMDAR-dependent physiologic and pathologic processes in the CNS are still unclear.

In the present study, we examined whether PSD-93 deficiency affected synaptic NR2A and NR2B expression in two major pain-related regions [18,19], spinal cord and forebrain cortex, and a motor and coordination-related region [20], cerebellum, of the CNS. Furthermore, we examined whether PSD-93 was required for NMDAR-dependent development of neuronal plasticity during morphine tolerance and physical dependence.

## Materials and methods

### Animals

The PSD-93 KO mice (C57BL/6 genetic background) were generated as described previously [21]. Male PSD-93 KO mice and wild type (WT) littermates (10–12 weeks) were obtained by interbreeding PSD-93 heterozygous mice. All animal experiments were carried out with the approval of the Animal Care and Use Committee at Johns Hopkins University. The experimenter was blind to the genotype of the mice in all studies.

### Tail-flick assay

A tail-flick apparatus (Model 33B Tail Flick Analgesy Meter, IITC Life Science, Woodland Hills, CA, USA) with a radiant heat source connected to an automatic timer was used to assess the analgesic response. A cut-off time latency of 10 s was used to avoid tissue damage to the tail. Tail-flick latencies were measured as the time required to induce a tail flick after applying radiant heat to the skin of the tail. The antinociceptive effects were expressed as the percentage of maximal possible analgesic effect (% MPAE): % MPAE = [(response latency - baseline latency)/(cut-off latency - baseline latency)] × 100%.

### Morphine-induced tolerance studies

Acute morphine tolerance was induced by two subcutaneous (s.c.) injections of 100 mg/kg morphine (WT: n = 10; KO: n = 10) or saline (control; WT: n = 10; KO: n = 10) given 12 h apart [22,23]; chronic morphine tolerance was induced by s.c. injections of morphine (10 mg/kg) (WT: n = 10; KO: n = 10) or saline (control; WT: n = 10; KO: n = 10) given every 12 h for 6 days [8]. On day 2 after induction of acute morphine tolerance or day 7 after induction of chronic morphine tolerance, cumulative dose-response curves were determined as described previously [24]. Mice received a very low morphine dose (1 mg/kg, s.c.) and analgesia was assessed 30 min later by the tail-flick assay. Mice that were not analgesic at the first dose then received a second dose (cumulative dosing increase by a 0.3 log unit) and were tested 30 min afterward. This procedure was repeated until either the mice did not move their tail within the cut-off time or no further increase in tail-flick latency was noted from one dose to the next.

To observe whether repeated morphine injection produced a time-dependent and NMDAR-dependent reduction in morphine analgesic effects, WT and KO mice received s.c. injections of morphine (10 mg/kg) or saline twice daily (12-h intervals) with intraperitoneal (i.p.) injection of saline or 0.3 mg/kg MK-801 once daily for 6 days. WT and KO mice were divided into four paired groups (n = 10 WT and 10 KO per group): s.c. saline + i.p. saline, s.c. morphine + i.p. saline, s.c. saline + i.p. MK-801, and s.c. morphine + i.p. MK-801. Tail-flick latencies were determined prior to drug injection and 1 h after the first injection of morphine on days 1, 3, 5, and 7.

### Morphine-induced abnormal pain hypersensitivity studies

To determine whether NMDAR-dependent mechanical allodynia occurs in morphine-tolerant mice, paw withdrawal response to mechanical stimuli was measured as described previously [16,25]. Mice received s.c. injections of morphine (20 mg/kg) or saline twice daily with i.p. injection of 0.3 mg/kg MK-801 or saline once daily for 6 days. WT and KO mice were divided into four paired groups (n = 6 WT and 6 KO per group): s.c. saline + i.p. saline, s.c. morphine + i.p. saline, s.c. saline + i.p. MK-801, and s.c. morphine + i.p. MK-801. Mechanical behavioral testing was performed before drug injection (baseline) and on days 1, 2, and, 4 after morphine withdrawal. Each mouse was placed in a Plexiglas chamber on an elevated mesh screen. Two calibrated von Frey filaments (0.24 and 1.47 mN; Stoelting Co., Wood Dale, IL, USA) were applied to the hind paw for approximately 1 s, and each stimulation was repeated 10 times to both hind paws. The occurrence of paw withdrawal in each of these 10 trials was expressed as a percent response frequency [(number of paw withdrawals/10 trials) × 100 = % response frequency], and this percentage was used as an indication of the amount of paw withdrawal.

To determine whether NMDAR-dependent thermal hyperalgesia occurs in morphine-tolerant mice, paw withdrawal response to noxious heat stimulation was measured as described previously [16,25]. WT (n = 24) and KO (n = 24) mice were divided into four paired groups and given drug treatments according to the same protocol as for the mechanical test. Thermal behavioral testing was carried out before drug injection (baseline) and on days 1, 2, and 4 after morphine withdrawal. Mice were placed in a Plexiglas chamber on a glass plate. A radiant heat from Model 336 Analgesic Meter (IITC Inc./Life Science Instruments, Woodland Hills, CA, USA) was applied by aiming a beam of light through a hole in the light box through the glass plate to the middle of the plantar surface of each hind paw. When the animal lifted its foot, the light beam was turned off. The length of time between the start of the light beam and the foot lift was defined as the paw withdrawal latency. Each trial was repeated five times at 5-min intervals for each side. A cut-off time of 20 s was used to avoid tissue damage to the hind paw.

The formalin test was performed as described previously [26]. Briefly, mice received s.c. injection of morphine (20 mg/kg) or saline twice daily for 6 days. On day 7, WT (n = 12; 6 for saline and 6 morphine) and KO (n = 12) mice received a 10-μl intraplantar injection of 1% formalin. After the formalin injection, the duration of paw licking was recorded in 5-min periods for 60 min. We defined the first phase response as the total time spent licking during the first 10 min and the second phase response as the duration of licking that occurred 10–60 min after formalin injection.

### Naloxone-precipitated withdrawal symptoms

Among naloxone-precipitated withdrawal symptoms, jumping is reliably observed in mice, although other symptoms, such as forepaw tremor and rearing, are sometimes observed [27]. To examine an NMDAR-dependent morphine physical dependence, the number of jumps was analyzed quantitatively. Briefly, WT and KO mice (10/genotype/treatment) were injected twice daily with morphine (10 mg/kg, i.p.) and once daily with 0.3 mg/kg MK-801 or saline for 6 days and on the seventh day challenged with naloxone (2 mg/kg, i.p.) 2 h after a final s.c. injection of morphine (10 mg/kg). Immediately after naloxone treatment, each mouse was placed into a transparent acrylic quadribox (20 × 30 cm), and jump frequency was tallied over the next 15 min.

### Subcellular fractionation of proteins

Biochemical fractionation was carried out according to previous studies with minor modification [28,29]. WT (n = 12) and KO (n = 12) mice were sacrificed and lumbar enlargement spinal cord, forebrain cortex, and cerebellum collected. The dorsal part of the spinal cord was separated from the ventral part. The tissues were homogenized in homogenization buffer [10 mM Tris-HCl (pH 7.4), 5 mM NaF, 1 mM sodium orthovanadate, 320 mM sucrose, 1 mM EDTA, 1 mM EGTA, 0.1 mM phenylmethylsulfonyl fluoride, 1 mM leupeptin, and 2 mM pepstatin A] and centrifuged at 1,000 × *g *for 20 min at 4°C. The supernatant (S1, total soluble fraction) was collected and the pellet (P1, nuclei and debris fraction) discarded. After measurement of the protein concentration, 20% of S1 was removed for detecting protein expression in the total soluble fraction. The remaining S1 (80%) was centrifuged at 10,000 × *g *for 20 min to produce a pellet (P2) and supernatant (S2). The P2 was lysed hypo-osmotically in water and centrifuged at 25,000 × *g *to produce pellet 3 (P3). The P3 was considered to be the crude synaptosomal membrane fraction [28,29].

### Western blot analysis

The samples were heated for 5 min at 99°C and then loaded onto 4% stacking/7.5% separating SDS-polyacrylamide gels. The proteins were eletrophoretically transferred onto nitrocellulose membrane. The blotting membrane was blocked with 3% non-fat dry milk for 1 h and incubated overnight at 4°C with rabbit anti-PSD-93 (1:1,000; Alomone Labs Ltd, Jerusalem, Israel), rabbit anti-*N*-cadherin (1: 1,000; BD Biosciences, Palo Alto, CA), rabbit anti-NR2A (1: 200, Upstate/CHEMICON, Temecula, CA), rabbit anti-NR2B (1:500, Upstate/CHEMICON), or monoclonal mouse anti-β-actin (1:10,000; Santa Cruz Biotechnology, Inc., Santa Cruz, CA). *N*-cadherin was used as a loading control and marker for crude synaptosomal fraction, whereas β-actin was used as a loading control for total soluble fraction. The proteins were detected with anti-rabbit or anti-mouse secondary antibody and visualized with the chemiluminescence reagents provided with the ECL kit (Amersham Pharmacia Biotech, Piscataway, NJ) and exposure to film. The intensity of blots was quantified with densitometry. The blot density from naïve animals was set as 100%.

### Statistical analysis

Data are expressed as mean ± SEM. The effective dose that resulted in a 50% reduction of control response (ED_50_) and 95% confidence intervals (CIs) were calculated by using a least-squares linear regression method according to the formulae given by Tallarida and Murray (1987). Statistical significance was determined by student's *t *tests or one-way and two-way analysis of variance followed by the post hoc Tukey tests. Significance was set at *P *< 0.05. The statistical software package SigmaStat (Systat, Port Richard, CA) was used to perform all statistical analyses.

## Results

### 3.1. Effect of PSD-93 deletion on the expression of NR2A and NR2B in total soluble and synaptosomal membrane fractions

**Figure 1:**
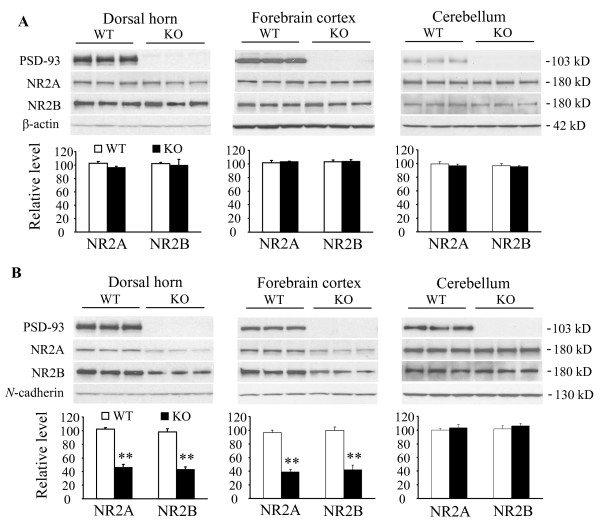
**Expression of NR2A and NR2B in total soluble (A) and synaptosomal membrane (B) fractions from dorsal horn, forebrain cortex, and cerebellum in wild type (WT) and PSD-93 knockout (KO) mice.** (A) Top: representative Western blots showing the levels of PSD-93, NR2A, and NR2B in the total soluble fraction. Bottom: statistical summary of the densitometric analysis expressed relative to the corresponding loading control (β-actin). (B) Top: representative Western blots showing the amounts of PSD-93, NR2A, and NR2B in the synaptosomal membrane fraction. Bottom: statistical summary of the densitometric analysis expressed relative to the corresponding loading control (*N*-cadherin). **P < 0.01 *vs *the corresponding naïve WT mice.

Immunoblot analysis showed that PSD-93 deletion did not alter expression of NR2A and NR2B in total soluble fractions from dorsal horn, forebrain cortex, or cerebellum of mice (Fig. 1A), a finding consistent with those in previous studies [16,21,30]. However, PSD-93 deletion significantly reduced the levels of NR2A and NR2B in the synaptosomal membrane fractions from dorsal horn and forebrain cortex, but not cerebellum, of mice (Fig. 1B). In the dorsal horn of KO mice, the amounts of NR2A and NR2B were decreased by 55% (*P *< 0.01) and 56% (*P *< 0.01), respectively, compared to those in WT mice. In the cortex of KO mice, the amounts of NR2A and NR2B were reduced by 60% (*P *< 0.01) and 58% (*P *< 0.01), respectively, compared to those in WT mice. In the cerebellum of KO mice, the levels of NR2A and NR2B were 103% (*P *> 0.05) and 104% (*P *> 0.05), respectively, of those in WT mice.

### 3.2. Effect of PSD-93 deletion on acute and chronic morphine tolerance

**Figure 2:**
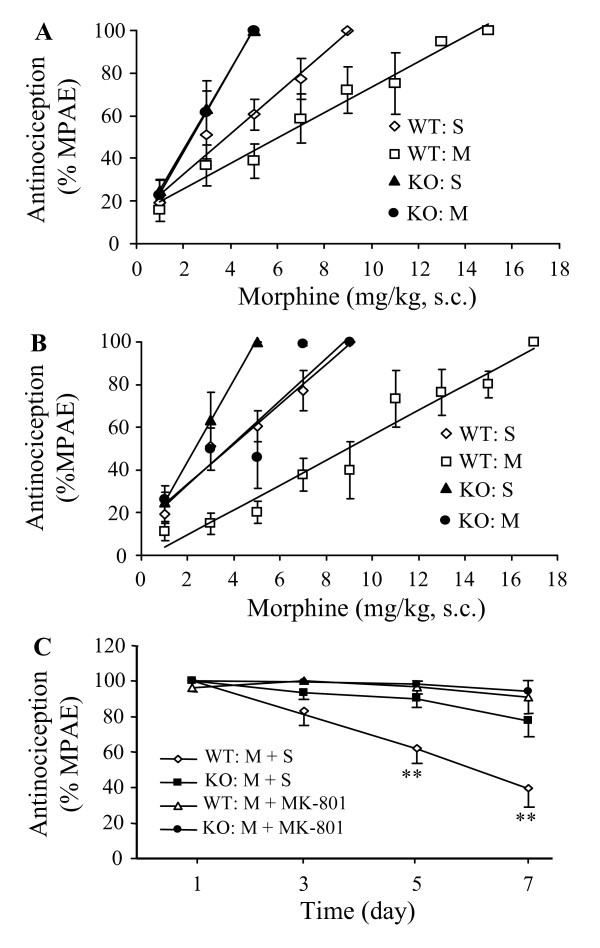
**Effect of targeted disruption of PSD-93 gene on morphine analgesic tolerance.** (A) The cumulative dose-response curves of morphine in WT and PSD-93 KO mice following acute morphine (M) analgesic tolerance induced by two subcutaneous injections of morphine (100 mg/kg, 12 h apart). Control groups received saline (S) injection on the same schedule. (B) The cumulative dose-response curves of morphine in WT and PSD-93 KO mice following chronic morphine analgesic tolerance induced by subcutaneous injections of morphine (10 mg/kg) twice daily for 6 days. Control groups received saline injections on the same schedule. (C) Time course of morphine-induced analgesia and effect of MK-801 in WT and PSD-93 KO mice following subcutaneous injections of 10 mg/kg morphine twice daily plus intraperitoneal injection of saline or 0.3 mg/kg MK-801 once daily for 6 days. ** *P *< 0.01 *vs *the corresponding value on day 1.

We first examined the role of PSD-93 in acute morphine tolerance. Baseline tail-flick latencies were similar in KO (4.84 ± 0.26; n = 19) and WT (4.40 ± 0.20; n = 20) mice (*P *> 0.05). In the control (saline-treated) groups, the ED_50 _of the dose-response curve of morphine in KO mice (ED_50_: 2.32 mg/kg; 95% CI: 1.89–2.99 mg/kg) was significantly lower than that in WT mice (ED_50_: 3.75 mg/kg; 95% CI: 2.87–5.43 mg/kg), although the analgesic effect induced by the first dose of morphine in KO mice was similar to that in WT mice (Fig. 2A and 2B). After acute morphine challenge, the morphine dose-response curve was shifted significantly to the right in the WT, but not KO, mice (Fig. 2A). The morphine ED50 value (and 95% CI) was 6.08 mg/kg (5.29–7.15 mg/kg) in the WT mice and 2.40 mg/kg (2.29–2.52 mg/kg) in the KO mice.

We then investigated the effect of PSD-93 deletion on chronic morphine tolerance. Chronic morphine challenge produced a rightward shift in the morphine dose-response curve in both WT and KO mice (Fig. 2B). However, the ED_50 _of the dose-response curve of morphine in KO mice was significantly smaller than that in WT mice (Fig. 2B). The morphine ED50 value (and 95% CI) was 8.92 mg/kg (7.47–11.08 mg/kg) in the WT mice and 3.55 mg/kg (2.05–13.05 mg/kg) in the KO mice. Chronic morphine challenge also produced a time-dependent reduction in morphine analgesic effects in the WT, but not KO, mice (Fig. 2C). In the WT mice that received repeated injections of s.c morphine plus i.p. saline, the MPAEs on days 3, 5, and 7 were reduced by 18.9% (*P *> 0.05), 38.3% (*P *< 0.01), and 60.2% (*P *< 0.01), respectively, from the value on day 1 (100%). These reductions were markedly reversed in the WT mice that received i.p. MK-801 rather than saline (Fig. 2C). In contrast, no significant changes in the MPAEs were observed among these four time points in the KO mice that received s.c. morphine plus i.p. saline or MK-801 (Fig. 2C). In addition, repeated injections of i.p. MK-801 at the dose used did not affect basal tail-flick latency in either the WT or KO mice that received s.c saline (data not shown).

### 3.3. Effect of PSD-93 deletion on morphine-induced abnormal pain hypersensitivity

**Figure 3:**
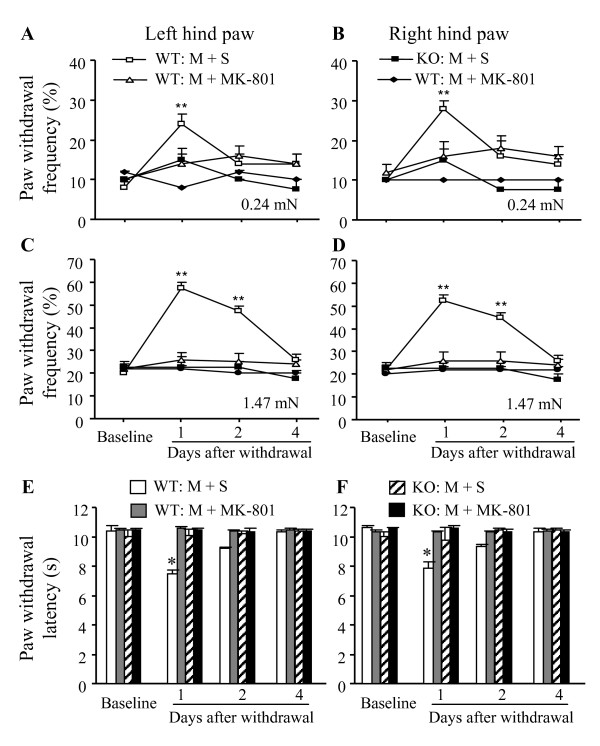
**Effect of targeted disruption of PSD-93 gene on mechanical allodynia and thermal hyperalgesia after repeated morphine injection.** WT and PSD-93 KO mice were injected twice daily with subcutaneous morphine (M, 20 mg/kg) and once daily with intraperitoneal saline (S) or 0.3 mg/kg MK-801 for 6 days. (A-D) Withdrawal responses of left (A and C) and right (B and D) hind paws to 0.24 mN (A and B) and 1.47 mN (C and D) intensity mechanical stimuli on days 1, 2, and 4 after morphine withdrawal. **P < 0.01 *vs *the corresponding baseline. (E and F) Withdrawal response of left (E) and right (F) hind paws to thermal stimulation on days 1, 2, and 4 after morphine withdrawal. *P < 0.05 *vs *the corresponding baseline.

Consistent with previous studies [31], mechanical allodynia and thermal hyperalgesia developed after morphine withdrawal in the WT mice that received repeated injections of s.c. morphine plus i.p. saline (Fig. 3). Paw withdrawal frequencies were significantly increased by 3 fold (*P *< 0.01) and 2.8 fold (*P *< 0.01) from baseline on left and right hind paws, respectively, in response to a low-intensity mechanical stimulation (0.24 mN) on day 1 after morphine withdrawal (Fig. 3A and 3B). Paw withdrawal frequencies were also markedly increased by 2.9 fold (*P *< 0.01) and 2.4 fold (*P *< 0.01) from baseline on left and right hind paws, respectively, on day 1 after morphine withdrawal and by 2.4 fold (*P *< 0.01) and 2.1 fold (*P *< 0.01) from baseline on left and right hind paws, respectively, on day 2 after morphine withdrawal in response to a moderate mechanical stimulation (1.47 mN; Fig. 3C and 3D). Paw withdrawal latencies in response to heat stimulation were significantly decreased by 27% (*P *< 0.05) and 32% (*P *< 0.05) from the baseline on left and right hind paws, respectively, on day 1 after morphine withdrawal (Figs. 3E and 3F). Both mechanical allodynia and thermal hyperalgesia were completely blocked in the WT mice that also received i.p. MK-801 (Figs. 3A–3F). In contrast, the KO mice that received repeated injections of s.c. morphine plus i.p. saline or MK-801 did not exhibit significant changes in paw withdrawal frequencies or latencies (Fig. 3). In addition, repeated injections of i.p. MK-801 at the dose used did not affect basal paw withdrawal frequency and latency in either WT or KO mice that received s.c saline (data not shown).

**Figure 4:**
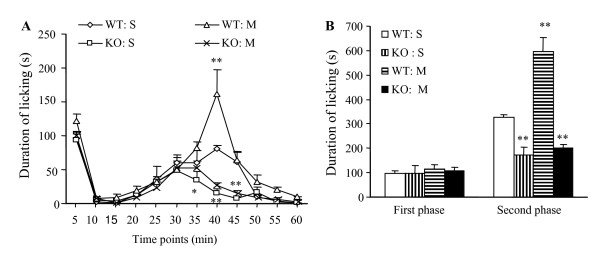
**Formalin-induced licking behaviors on day 1 after morphine withdrawal in WT and KO mice that received subcutaneous injections of 20 mg/kg morphine (M) or saline (S) twice daily for 6 days.** (A) Time course of formalin-induced licking behaviors. *P < 0.05 or **P < 0.01 *vs *the values in the saline-treated WT mice at the corresponding time point. (B) Summary of licking duration in the first and second phases. **P < 0.01 *vs *the saline-treated WT mice in the second phase.

We also examined whether PSD-93 deletion affected morphine-induced behavioral sensitivity in response to formalin-induced noxious inflammation [26,31]. As expected, an intraplantar injection of 1% formalin produced characteristic biphasic licking behaviors in the mice (Fig. 4A). The first phase behavioral response of the formalin test was similar in all groups of WT and KO mice (Fig. 4). The second phase response was greatly enhanced in the morphine-treated WT mice (Fig. 4). Duration of paw licking was increased by 1.8 fold of the value in the saline-treated WT mice (*P *< 0.01). However, durations of paw licking in the saline- and morphine-treated KO mice were significantly reduced by 47% (*P *< 0.01) and 39% (*P *< 0.01), respectively, compared to that in the saline-treated WT mice. No significant difference was observed in the second phase response between the saline- and morphine-treated KO mice (Fig. 4).

### 3.4. Effect of PSD-93 deletion on naloxone-induced withdrawal symptoms

**Figure 5:**
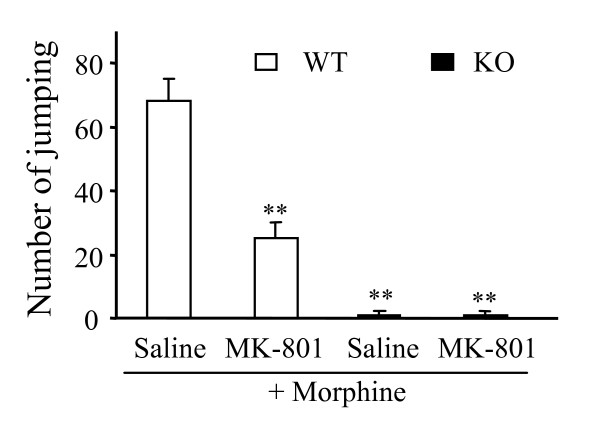
**Effect of targeted disruption of PSD-93 gene on physical dependence induced by repeated morphine injections.** WT and KO mice were given morphine injections (10 mg/kg, s.c.) twice daily for 6 days plus a once-daily intraperitoneal injection of either saline or 0.3 mg/kg MK-801. Bars represent mean number of jumps in the 15-min period following a single naloxone injection (2 mg/kg) that was given 2 h after a final 10-mg/kg subcutaneous morphine injection. **P < 0.01 *vs *the saline + morphine group.

Consistent with previous studies [8,27], repeated injections of s.c. morphine plus i.p. saline produced striking jumping activity, one of naloxone-precipitated withdrawal symptoms, in the WT mice (Fig. 5). This jumping activity was significantly blocked in the WT mice that received s.c. morphine plus i.p. MK-801 (*P *< 0.01; Fig. 5). The KO mice that received repeated injections of s.c. morphine plus i.p. saline or MK-801 jumped significantly less than the WT mice (Fig. 5). The number of jumps was 98.5% less in the KO than in the WT mice that received s.c morphine plus i.p. saline (*P *< 0.01).

## Discussion

An important observation in the present study is that PSD-93 deficiency produces distinct effects on synaptic NMDAR expression in different regions of the CNS. Evidence from *in vitro *studies shows that PSD-93 clusters and anchors NMDARs at synapses through interaction of its PDZ domains with seven C-terminal amino acids of NR2A and NR2B [9,10]. Our quantitative Western blot analysis showed that PSD-93 deficiency significantly reduced the levels of NR2A and NR2B proteins in the synaptosomal membrane fractions of dorsal horn and forebrain cortex but did not affect their expression in the total soluble fractions of these two regions. This finding is consistent with our previous *in vitro *study, which showed that surface NR2A and NR2B expression is dramatically reduced in cultured dorsal horn neurons of KO mice [16]. The data suggest that PSD-93 is required for synaptic expression and localization of NR2A and NR2B in dorsal horn and forebrain cortex. However, PSD-93 deficiency did not markedly change the amounts of NR2A and NR2B in either synaptosomal or total soluble fractions from cerebellum. Hippocampus neurons in PSD-93 KO mice may also express normal levels of NR2A and NR2B at synapses because PSD-93 deletion does not alter NMDAR-mediated excitatory postsynaptic currents and potentials in hippocampus neurons [17]. It appears that the effect of PSD-93 deletion on NR2A and NR2B synaptic expression may be tissue specific in the CNS, although the detailed mechanisms underlying these events are still unclear. PSD-95 and SAP102, two members of the MAGUK family of proteins, also interact with NR2A and NR2B [13,32]. It is very likely that PSD-95 and SAP102 compensate for the deficiency of PSD-93 to anchor and target NMDARs at synapses in hippocampus and cerebellum neurons of PSD-93 KO mice.

Spinal cord and forebrain cortex are two major pain-related regions in the CNS [18,19]. Our previous studies demonstrated that PSD-93, NR2A, and NR2B are highly expressed in the superficial dorsal horn of spinal cord [16]. Under electron microscopy, the sections from superficial dorsal horn and the anterior cingular cortex of forebrain exhibited double labeling for PSD-93 and NR2A/NR2B in the postsynaptic density [16]. Furthermore, by co-immunoprecipitation, we found that both NR2A and NR2B were immunopreciptated by PSD-93 antibody in the postsynaptic density fractions of the spinal cord and forebrain cortex [16]. These findings demonstrate PSD-93 binding to NMDARs in the spinal cord and forebrain cortex *in vivo*. Electrophysiological recordings showed that PSD-93 deletion reduced NMDAR-mediated postsynaptic responses in these two regions of adult mice [16]. Behavioral study revealed that mice lacking PSD-93 exhibited blunted NMDAR-dependent persistent pain induced by peripheral nerve injury or injection of Complete Freund's Adjuvant, although they displayed intact nociceptive responsiveness to acute pain [16]. The present study showed that PSD-93 deficiency significantly inhibited acute and chronic morphine analgesic tolerance, enhancing formalin-induced pain behaviors, and withdrawal-induced jumping following repeated morphine injection. Morphine-induced tolerance, abnormal pain hypersensitivity, and physical dependence are considered to be related to NMDAR-dependent neuronal plasticity in the CNS [6-8]. Thus, it is very likely that these impaired morphine analgesic tolerance and physical dependence in PSD-93 KO mice is attributed to PSD-93 deletion-induced reduction in postsynaptic expression and function of NMDARs in dorsal horn and forebrain cortex neurons. Hippocampal NMDARs play a critical role in neuronal plasticity underlying learning and memory [33], whereas cerebellar NMDARs are involved in the modulation of motor learning and coordination [20]. Because PSD-93 deletion does not affect synaptic NMDAR expression and NMDAR-mediated postsynaptic functions in these two non-pain-related regions [17], PSD-93 deletion does not produce hippocampal and cerebellar dysfunctions, but does attenuate the development of persistent pain and morphine tolerance and physical dependence. Thus, targeted disruption of PSD-93 or perturbing NMDAR-PSD-93 interaction might be a better strategy for prevention and/or treatment of persistent pain and opioid tolerance and physical dependence in clinic.

It should be noted that PSD-93 also functions as a scaffolding protein to assemble a specific set of signaling proteins around the NMDARs. These signaling proteins, such as neuronal nitric oxide (NO) synthase (nNOS), participate in downstream signaling by the NMDARs. The PDZ domain of nNOS interacts with the PDZ domains of PSD-93 [9]. Deleting the PDZ domain from PSD-93 reduces NOS activity [9]. Our previous study showed that targeted disruption of PSD-93 gene significantly attenuates the NMDA-stimulated increase in cyclic guanosine 3', 5'-monophosphate in the cultured forebrain cortex neurons [30]. Given that inhibition of nNOS attenuates the development of persistent pain and morphine analgesic tolerance and physical dependence [34,35], it is very likely that the dissociation of NMDARs from NO signaling caused by PSD-93 deletion is also involved in the mechanism underlying impairing persistent pain and morphine analgesic tolerance and physical dependence in the KO mice. Besides NMDARs and nNOS, PSD-93 binds to other postsynaptic membrane proteins, such as potassium channels [9,10], δ_2 _glutamate receptors [36], and the microtubule-associated protein 1A [36,37]. Therefore, the detailed mechanism through which PSD-93 deficiency affects the development of neuronal plasticity in persistent pain and morphine tolerance and physical dependence remains to be explored.

In conclusion, the present study was the first to demonstrate that PSD-93 deletion has distinct effects on synaptic NMDAR expression in the CNS. Blunted NMDAR-dependent neuronal plasticity following repeated morphine injection in PSD-93 KO mice is attributed to PSD-93 deletion-induced alterations of NR2A and NR2B postsynaptic expression in dorsal horn and forebrain cortex neurons, but not in cerebellar neurons. Our findings suggest that PSD-93 might be a potential biochemical target for the treatment of opioid tolerance and physical dependence.

## Competing interests

The authors declare that they have no competing interests.
